# Broad-Scale Climate Influences on Spring-Spawning Herring (*Clupea harengus*, L.) Recruitment in the Western Baltic Sea

**DOI:** 10.1371/journal.pone.0087525

**Published:** 2014-02-25

**Authors:** Joachim P. Gröger, Hans-Harald Hinrichsen, Patrick Polte

**Affiliations:** 1 Thünen Institute of Sea Fisheries, Hamburg, Germany; 2 Institute for Bio-Sciences, Rostock University, Rostock, Germany; 3 GEOMAR Helmholtz Centre for Ocean Research, Kiel, Germany; 4 Thünen Institute of Baltic Sea Fisheries, Rostock, Germany; Aristotle University of Thessaloniki, Greece

## Abstract

Climate forcing in complex ecosystems can have profound implications for ecosystem sustainability and may thus challenge a precautionary ecosystem management. Climatic influences documented to affect various ecological functions on a global scale, may themselves be observed on quantitative or qualitative scales including regime shifts in complex marine ecosystems. This study investigates the potential climatic impact on the reproduction success of spring-spawning herring (*Clupea harengus*) in the Western Baltic Sea (WBSS herring). To test for climate effects on reproduction success, the regionally determined and scientifically well-documented spawning grounds of WBSS herring represent an ideal model system. Climate effects on herring reproduction were investigated using two global indices of atmospheric variability and sea surface temperature, represented by the North Atlantic Oscillation (NAO) and the Atlantic Multi-decadal Oscillation (AMO), respectively, and the Baltic Sea Index (BSI) which is a regional-scale atmospheric index for the Baltic Sea. Moreover, we combined a traditional approach with modern time series analysis based on a recruitment model connecting parental population components with reproduction success. Generalized transfer functions (ARIMAX models) allowed evaluating the dynamic nature of exogenous climate processes interacting with the endogenous recruitment process. Using different model selection criteria our results reveal that in contrast to NAO and AMO, the BSI shows a significant positive but delayed signal on the annual dynamics of herring recruitment. The westward influence of the Siberian high is considered strongly suppressing the influence of the NAO in this area leading to a higher explanatory power of the BSI reflecting the atmospheric pressure regime on a North-South transect between Oslo, Norway and Szczecin, Poland. We suggest incorporating climate-induced effects into stock and risk assessments and management strategies as part of the EU ecosystem approach to support sustainable herring fisheries in the Western Baltic Sea.

## Introduction

The EU Marine Strategy Framework Directive and the recently revised EU Common Fisheries Policy requires the development of sustainable ecosystem-based management strategies to reach the goal of Good Environmental Status. A key objective in European fishery management thereby is to provide the greatest societal benefit on a sustainable and precautionary basis. Subject to different constraints, this can be interpreted in different ways. One important constraint is the limited understanding of highly fluctuating recruitment processes of exploited fish populations and how they interact with exogenous factors.

A major problem in assessing fish populations is that ecosystem effects are most often ignored when fisheries advice is formulated. However, it is known that major limitations are essentially triggered by the environment. One important limit set by latent factors is the carrying capacity of the ecosystem. This complex of limiting factors, which is most likely also the shaping source of density dependence, can by itself be considered predominately driven by global-scale forces such as climate. Hence, climate related changes in complex ecosystems can have profound implications for ecosystem sustainability in many ways and may challenge a precautionary ecosystem management. Taking into account climate forcing in models may thus significantly reduce the degree of non-explained ecologically induced variation and as such lowers predictive certainty.

Landings of Western Baltic Sea (WBSS) herring (*Clupea harengus*, L.) have declined substantially over the last decade. These declines have been linked to intensive exploitation, but the role of environmental conditions along with overharvesting is not satisfyingly understood and hence cannot be neglected. How climate-related changes in the system would affect WBSS herring is presently unknown. Mechanisms could include a combination of climate-induced changes in hydrographical features interacting with ecological variables such as predator-prey relationships. From the perspective of the authors, linkages between WBSS population dynamics and environmental cycles need to be explored on a global scale allowing for the reproduction of emergent properties of the system such as regime shifts, and secondly facilitating the implementation of results into assessment and management procedures. Accordingly, the biological hypotheses included in this study are:

- a significant correspondence exists between global climate and WBSS herring with one or more clear climate signals to be identified; a qualitative correspondence may also be inherent when comparing shift patterns
- Climate-forced changes will predominately affect the earliest ontogenetic stages of WBSS herring, as documented for other species in different parts of the world [1], [2].

Integrating these aspects via extended stock-recruitment models into stock assessment models would not only facilitate modern ecosystem approaches, but can be expected to significantly improve management procedures.

## Materials and Methods

### Biological Data

**Figure 1:**
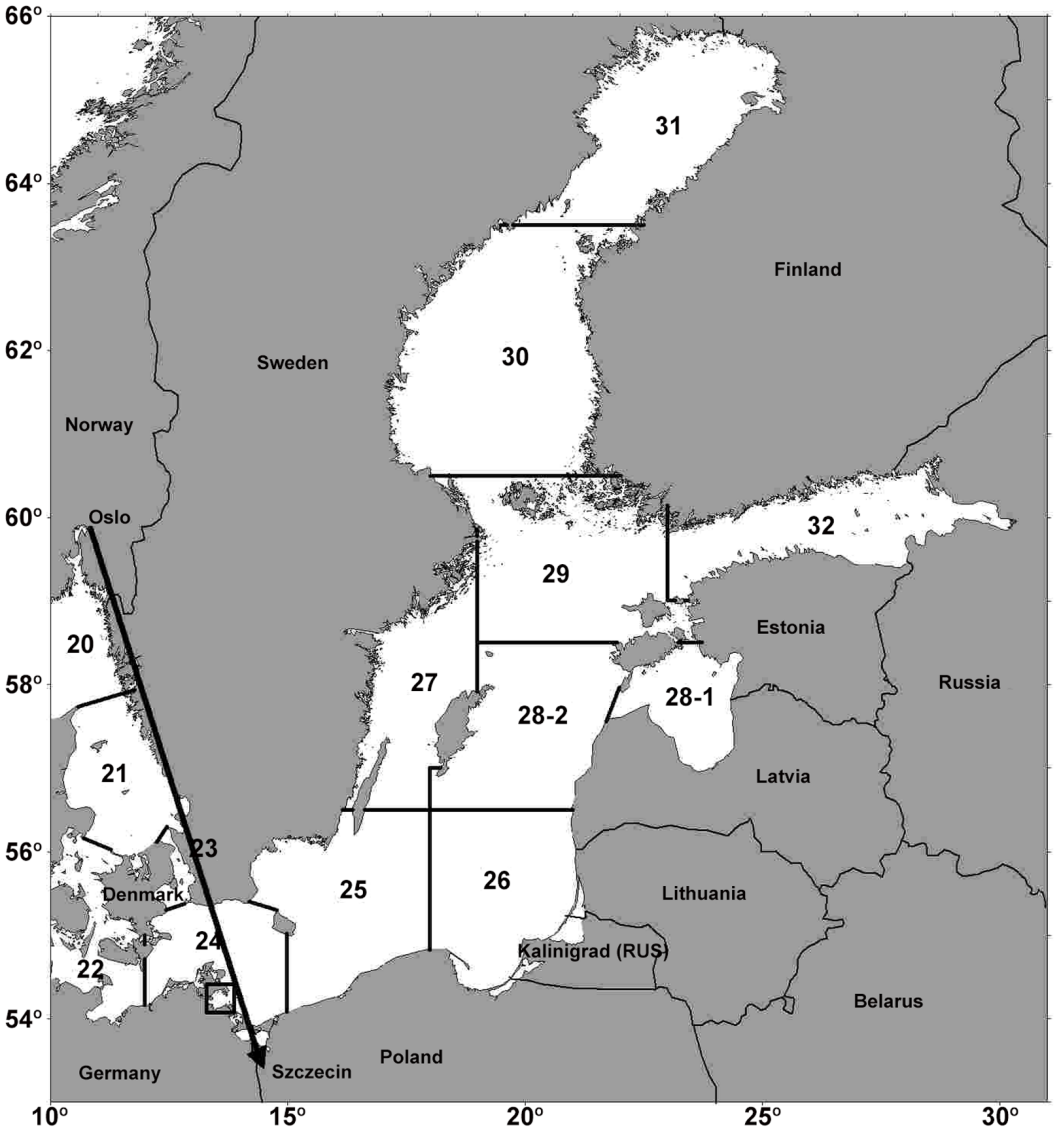
Map of the Baltic Sea area surrounded by neighbouring countries. The inset shows the location of the study area (south-eastern coast of Rügen Island plus the Greifswalder Bodden). The straight broken line connects Oslo (Norway) with Szczecin (Poland) representing the direct geographical distance between the two locations the BSI atmospheric pressure index has been calculated for.

Greifswald Bay in the German part of the South-Western Baltic Sea is considered as a major spawning area of WBSS herring (Figure 1). After early juvenile stages are spent close to shore, the 2+ *wr* group (individuals with two or more winter rings in their otoliths) migrate out of the Rügen area during the 2^nd^ quarter of the year, to feed in the Kattegat and Skagerrak area (ICES–Management Division IIIa) as well as in the neighbouring North Sea. Between February and May herring returns to the Western Baltic Sea for spawning [3], [4], [5], [6]. On Skagerrak and Kattegat feeding grounds WBSS herring overlaps with North Sea autumn-spawning stocks introducing the requirement to split the stocks for reliable assessments.

**Figure 2:**
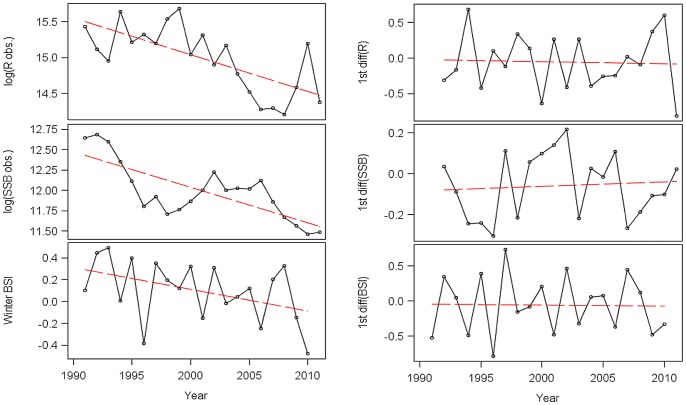
Plots of the dataseries of log_e_(*R*), log_e_(SSB), and winter BSI from 1991 to 2011, where *R* is WBSS herring recruitment, SSB is spawning-stock biomass for WBSS herring, and BSI the Baltic Sea index. Left panels: Non-stationary (original) dataseries (open dots connected by solid lines) with significant linear trend lines (broken lines). Right panels: stationary dataseries, with lines as in the three left panels, and values distributed around zero (detrended by taking 1^st^ order differences).

Estimates of WBSS herring spawning-stock biomass (SSB) and recruitment (*R*, age 1) were obtained from the last report of the International Council for the Exploration of the Sea (ICES) Herring Assessment Working Group [7], based on virtual population estimates of population size and mortality rates for the period 1991–2011 (Figure 2). These estimates integrate information derived from commercial and recreational catch-at-age data, discards, and fishery-independent research surveys for the region. Details on data sources and analytical methods employed in stock assessment are provided in [7]. However, given the requirements of “autoregressive integrated moving average” (ARIMA) modelling procedures (see below for details), and because the relevant data series do not appear mean stationary (Figure 2) during the period under consideration (1991–2011), the data series were first made stationary (Figure 2) before being used for further analyses.

### Climate Data

Broad-scale climate indices were used to represent large-scale processes that may influence the recruitment of fish stocks in the North Atlantic Ocean: the Baltic Sea Index (BSI), the North Atlantic Oscillation (NAO), and the Atlantic Multidecadal Oscillation (AMO). These standardized climate proxies are considered as latent factors that represent more regionalized effects that are difficult to compare individually and inter regionally. Such characteristics help to avoid problems generated by redundancy, multi co-linearity or error inflation [8]. While both the BSI and the NAO are atmospheric sea level pressure anomalies (SLP), the AMO is an index of long-term sea surface temperature (SST) in the North Atlantic Ocean [9]. Whereas the NAO is based on differences in the normalized SLP between Iceland and either the Azores or Portugal [10], [11], [12], the BSI is based on differences in normalized SLPs between Oslo (Norway) and Szchezin (Poland). Because those station based indices are fixed in space while the NAO centers move throughout the annual cycle such indices can only adequately capture NAO variability for parts of the year [13]. Accordingly, we used an averaged NAO index over the period December–March (winter NAO), covering the beginning of the WBSS herring spawning season. For reasons of comparability, we also used the BSI index averaged over the same period to give a winter BSI. As the temporal pattern of AMO does not differ by month, the annual averages were used as SST index. For details regarding the NAO index, see [13], [14], for those of the BSI, see [15], [16], [17] and for those of the AMO index, see [9] and http://www.esrl.noaa.gov/psd/data/timeseries/AMO/. Given the constraints of ARIMA modelling procedures (see below) all data series are required to be stationary (Figure 2b, 4th panel). To capture the characteristics of the entire climate time-series including long-term cycles, the full BSI, NAO and AMO time-series were used for pre-whitening and also for deriving a generalized transfer function (ARIMAX; for both procedures see below).

### Statistical Hypotheses Testing using a Climate-extended Stock-recruitment Model

In contrast to parental fish stocks, that tend to be rather influenced by biological factors, early life stages tend to be predominately influenced by their ambient physical environment. The survival success of passive egg and larval stages may be considered evolutionarily adjusted to prevailing hydrographic processes, synchronised with food provision (quantitatively plus qualitatively). We may assume cascadal effects ranging from global- to small-scale levels in the following hierarchical manner: climate → abiotic environment → biotic environment → eggs → larvae → recruitment. Hence, higher order levels trigger lower levels via the cascadal flow. It is obvious that this cascadal structure contains indirect effects that may in addition be delayed, simply because of the long “signal travelling time”. Accordingly the underlying hypothesis of this study is that the reproduction of Baltic Sea herring will in particular be most likely affected by abiotic changes in the ambient environment, triggered by strong changes in the climatic patterns. Statistically it makes sense to consider climate as higher order exogenous variable in modelling approaches functioning as latent background factor. The advantage of using one latent factor instead of multiple environmental (e.g. hydrographic) variables or products of variable aggregation methods (such as principal component analysis, factor analysis or variable clustering) is as follows: in contrast to the second option a latent factor contains already a large portion of relevant process information in a condensed manner, which is normally spread over many variables. This avoids all the problems generated through multiple factor inclusion such as redundancy or multi-collinearity induced by an unknown level of variable interaction, unknown causal structures between variables, unwanted reduction of the degrees of freedom, complex and/or non-reversal transformations, etc. Thus in summary recruitment may be seen as a function of the parental stock, which initially generates the spawning products, plus climatic factors that take over the abiotic cascadal control during the development of the external stages (i.e. egg and larvae stages). Statistically these influences can be tested by setting up the following statistical hypotheses:

H_0_: no climate effect, no stock size effect, no climate/parent stock interaction effect

H_1_: climate effect and/or stock size effect.

These hypotheses can either be tested using classical parameter tests (significance tests) and/or by using information criteria (see below) that quantify the performance of a model and thus aid in variable selection. The climate/parental stock interaction effect needs to be tested, because of a potential redundancy problem (multi-collinearity), and to avoid misinterpreting the climate signal as direct signal although it may have been (at least partially) mediated via the parental stock. However, as these effects may also be delayed we need to additionally test the significance of lagged climate signals and that of lagged parental stock effects using cross-correlation analysis (see below).

**Figure 3:**
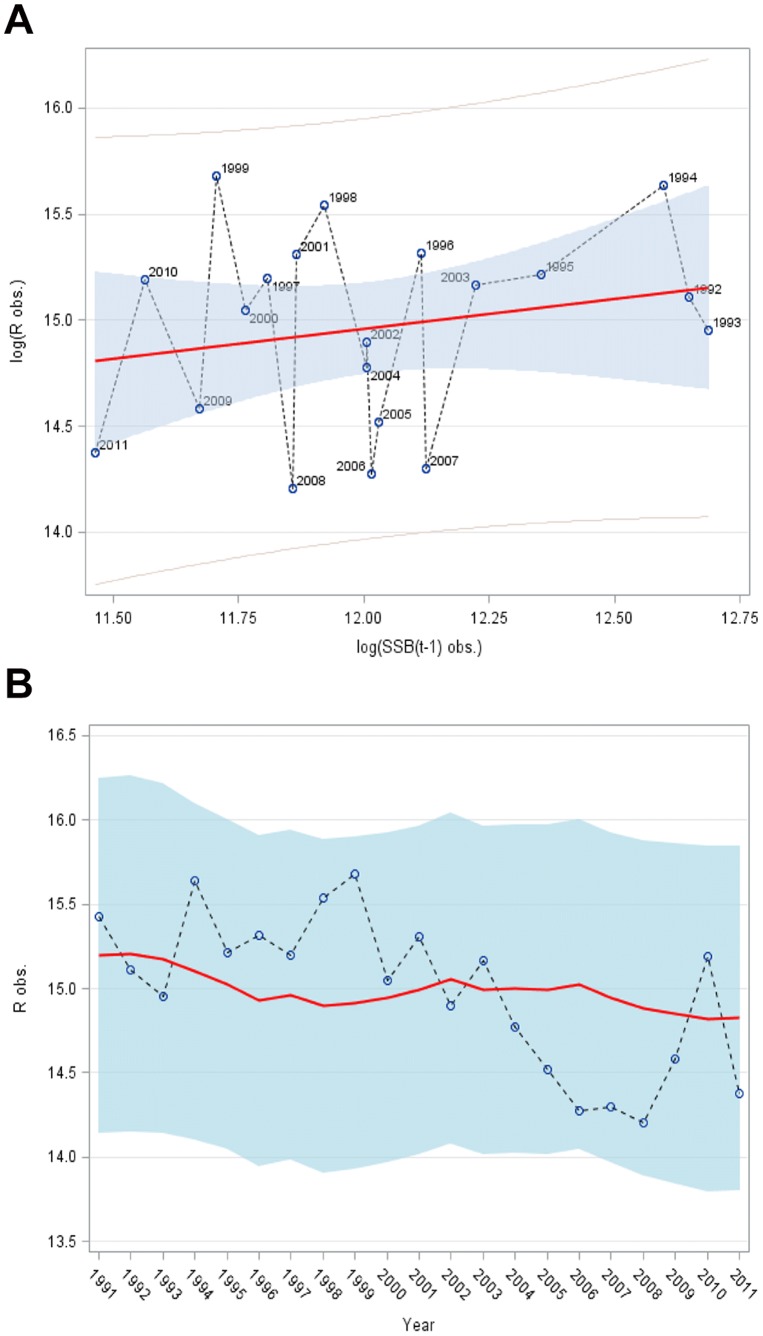
Cushing model [Equation (1) – see text] fitted to Rügen herring recruitment data. (A) Plot of log_e_ recruitment *R* (numbers in log_e_-thousands) against log_e_ spawning-stock biomass SSB (log_e_-t); the continuous line represents the predicted values of log_e_ *R* based on the Cushing model, the open dots connected by a dotted line and annotated by year represent observed log_e_ values of *R* at observed log_e_ SSB, and the light blue area the 95% prediction interval related to the Cushing model. (B) Plot of log_e_ *R* over time (years); the continuous line represents the predicted values of log_e_ *R* based on the Cushing model, the open dots the observed log_e_ values of *R*, and the light blue area the 95% prediction interval related to the Cushing model.

The best way to address the above setup of hypotheses is to start with some model formulation, in our case with a simple Cushing-type stock–recruitment model [18] which provides a baseline (deterministic part only):

 (1)where *R* is recruitment at age 1, SSB the spawning-stock biomass of the parental stock, and *α* and *δ* are model parameters. This is equivalent to imposing a single structural constraint in the model to reflect the functional relationship between WBSS herring recruitment at age 1 in year *t* and the SSB in year *t*–1 that produced these recruits. Figure 3 is a two-panel diagram depicting the fit of the Cushing-type stock–recruitment model to the WBSS herring recruitment data.

We next expanded this basic model to consider the *ad hoc* selected climate covariates attempting to explain more of the variance in recruitment (deterministic part only):

 (2)where *γ_1_*, *γ_2_* and *γ_3_* are coefficients associated with the BSI, the NAO and the AMO, l a time-lag for the effect of the BSI on recruitment, *k* a time-lag for the effect of the NAO on recruitment, *h* a time-lag for the effect of the AMO on recruitment, and all other terms are defined as before. The identification of the final model structure as well as addressing the question which of the exogenous variables (SSB, NAO, BSI, AMO) finally to include, at what lags (h, k, l) and with what parameter values to be estimated is subject to an explorative model (variable) selection procedure, among others based on cross-correlations using different performance measures (see below). This model structure however implies that the climate factors have a multiplicative effect on recruitment. The model in equation (2) can be linearized by taking natural logarithms of both sides to give (deterministic part only)

 (3)where all the terms are as defined above. This definition however implies Granger causality (see below).

In recognition of the time-series (TS) nature of the observations, we cast the estimation problem as a multiple TS analysis. For early applications of TS analysis to stock–recruitment data, see [19], [20]. The full transfer function model (i. e. the model including the error structure *η_t_*) for this problem can be expressed as.

 (4)where *B* is a so-called backshift operator of type *B*X_t_ = x_t-1_, *B* ^2^X_t_ = x_t-2_, …, *B* ^k^X_t_ = x_t-k_ specifying appropriate time-delays 1, 2, …, k for the variables’ linear combinations, *η_t_* a random error term that can be modelled as an autoregressive, integrated-moving-average process and all other terms are as defined before. In our analysis, the time delay is fixed at 1 for the log_e_(SSB*_t_*) term, and the time delays in terms of *l*, *k*, and *h* for BSI, NAO and AMO need to be determined empirically. The transfer function model permits a much more general error structure than an ordinary least squares regression model specified for the same set of observations. It also imposes important constraints on the stationarity of the series (see below).

For the transfer-function model, we based part of our statistical treatment on methods described by [21], [22] for developing multiple TS models. The development of transfer-function models is typically based on empirical patterns in the cross-correlation functions (CCFs) between the input variables and the output variable, and on patterns of autocorrelation (ACF) and partial autocorrelation (PACF) in the residuals of the model (Table 1). Cross-correlation analysis is one of the most essential tools to study Granger causality [23] which basically states that only predetermined (past) values of the same (endogenous variable, output) or another time series variable (exogenous variable, input) can have an influence on future values, but future values not on past values. Thus Granger causality defines causal direction through the temporal order of the underlying TS values.

**Table 1:** General pattern of the autocorrelation function (ACF) and the partial autocorrelation function (PACF) relative to the type of process, where AR is the autoregressive component of order *p* of the process, MA the moving average component of order *q* of the process, and ARMA a combination of both.

Component	ACF	PACF
**AR**	Tails off exponentially or in sine-waves	Drops off after lag p
**MA**	Drops off after lag *q*	Tails off exponentially or in sine waves
**ARMA**	Tails off exponentially or in sine-waves	Tails off exponentially or in sine waves

**Figure 4:**
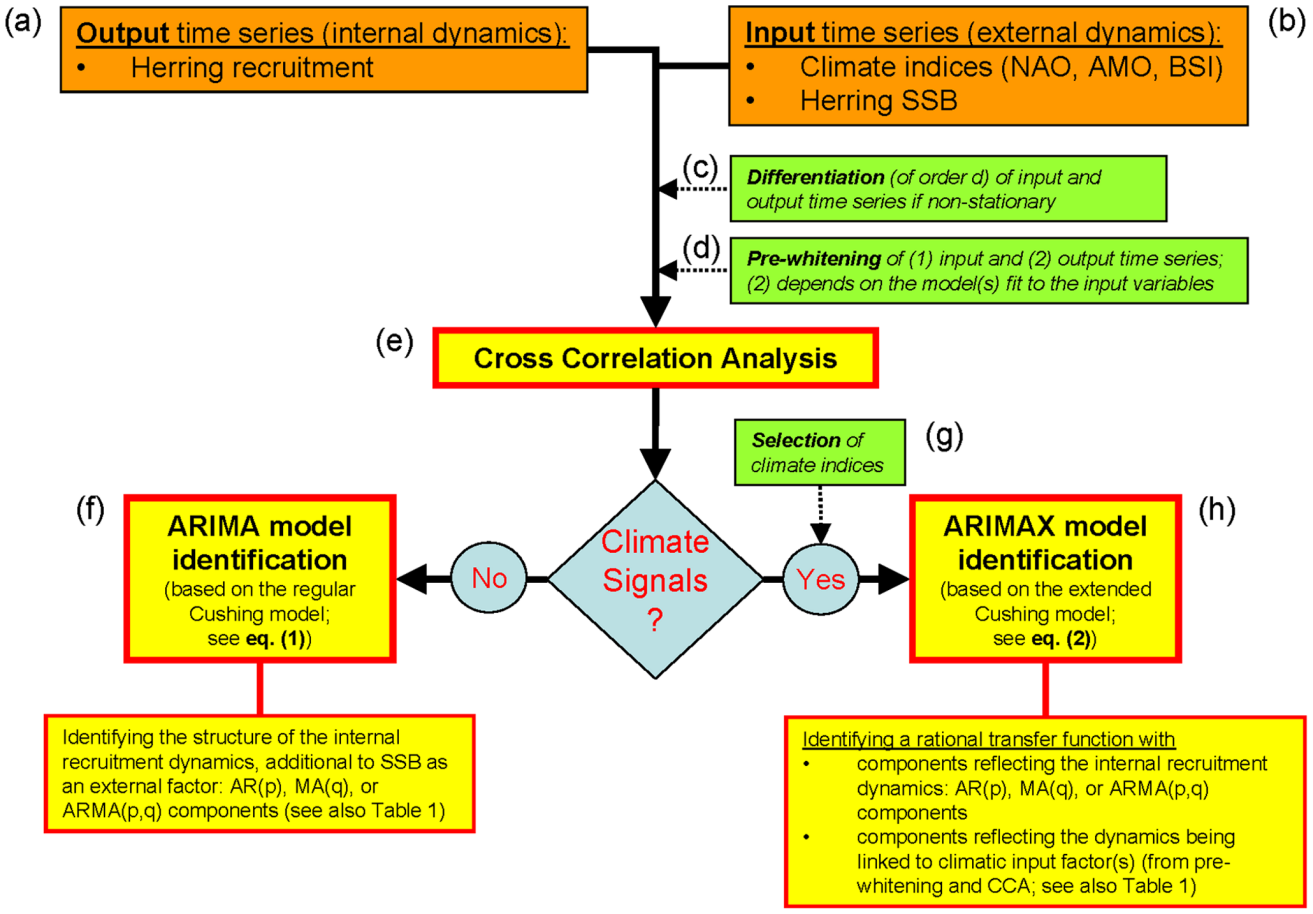
Conceptual illustration of the variable and model selection algorithm.

Figure 4 conceptually summarizes and illustrates the entire variable and model selection algorithm, respectively. This type of analysis basically tries to identify and estimate the structure and order of an underlying autoregressive integrated moving average (ARIMA) process based on stationary TS (Figure 4a, c, f). If this process is further linked to exogenous variables (*X*), as in our case, then it is referred to as an ARIMAX process or a transfer function (Figure 4a–e, g–h). As the variables in our analysis were non-stationary, we first took differences [21] of each variable (Figure 4c). Thus, in contrast to the approach taken in traditional regression models, we modelled dynamic change in the processes. To guard against identifying spurious relationships, univariate TS models were then developed for the input variables (BSI, NAO, AMO) and thereafter used to reduce both the input and output series to white noise (Figure 4d). The filtered (or “pre-whitened”) series were then cross-correlated to identify the appropriate model structure (Figure 4e).

As noted above, we imposed a single structural constraint in the model to reflect the functional relationship between WBSS herring recruitment at age 1 in year *t* and the SSB in year *t*–1 that produced these recruits. The estimation and model-selection procedure can be subdivided into five principal steps (Figure 4f–h):

- identification of model structure according to the behaviour of the autocorrelation (ACF) and partial autocorrelation functions (PACF), as given in Table 1;
- estimation of model parameters;
- diagnostic checking of model residuals;
- diagnostic forecasting (cross-validation);
- real prognosis,

with steps (iv) and (v) being outside the real modelling phase and hence being ignored here. For details on performing these steps in a marine biological context, see [24], [25], [26]. For a purely statistical description, see [21], [27], [28], [29].

### Performing Cross-correlations and Model Diagnostics

In contrast to simple correlation, CCFs require two treatments of the data before they can be cross-correlated to avoid bias: (i) both time-series need to be made stationary (in both mean and variance), and (ii) the risk of identifying spurious correlations must be minimized by then pre-whitening the data. These two treatments change the association of the two variables to be cross-correlated, implying that the results from cross-correlations cannot be compared with those from simple correlation. The first step is intended to detrend the data to achieve stationarity in the mean and to make them homoscedastic (stationary in variance), two prerequisites for ARIMA modelling.

All time-series were differenced, converting the data from absolute values to sequential changes in time (rates). As the intended ARIMAX model uses log_e_-transformed estimates of recruitment as output as well as log_e_-transformed estimates of SSB, annual AMO, winter NAO and winter BSI data as input variables, these variables were ensured stationary in mean and variance through selection of the right order of differentiation. To check this, mean stationarity was tested by testing the slope of a linear trend under the null hypotheses that the slope differs from zero, variance stationarity was tested using the Levene’s test under the null hypothesis of homoscedasticity.

The second step involves first fitting an ARIMA model to each input variable, separately applying each of these models to the output variable, then using the residuals of each fitted model to cross-correlate them separately with the output variable. This is necessary to preclude false signals being generated that can result simply from concurrent similar trends and the sequential order of the data that do not reflect a true underlying relationship among the two variables. To capture the full cyclic pattern of all input (exogenous) variables, this part of the analysis was based on the entire exogenous time-series, in the case of the winter NAO going back to 1821, in the case of winter BSI back to 1970, and in the case of the all-year AMO back to 1856.

To find the best model all permutations of ARIMAX terms (auto-regressive (AR) terms, moving average (MA) terms, SSB, and climate parameter specifications) plus the model assumptions need to be tested using standard model-selection procedures. As it is good scientific practice to use more than one criterion we made use of cross-correlations, of two information criteria (including Akaike’s Information Criterion (AICC) [30] bias corrected for small samples [31], [32], [33] plus Schwartz/Bayes Criterion (SBC) [34], as well as of residual diagnostics (including Ljung/Box tests i.e. Chi^2^-based autocorrelation checks of residuals, also called Portmanteau tests, for small TS under the null hypothesis of no residual autocorrelation). As a qualitative guide to assess model performance we estimated r_performance_ as the coefficient of correlation between predicted and observed values for each model [26] giving values that can range from 0 (worst) to 1 (best). We generally set our significance level to *α* = 0.05. For distributional tests of normality as well as Levene’s tests on homoscedasticity, a higher *α* (0.1) was selected, to increase the power (1–*β*; by decreasing the type II error *β*) and reduce the risk of falsely accepting the null hypothesis of either the residuals being normally distributed or the TS being homoscedastic, respectively, if they are not [35]. We used SAS 9.3 to perform all analyses.

To avoid misinterpretation, it should be noted that CCFs cannot be compared with Pearson’s correlation coefficients or with *r*_performance_ measures. Such a comparison would be misleading because the apparent levels of significance of the latter two may be artificially inflated by serial correlation, reducing the effective degrees of freedom [36]. In contrast, CCFs are based on stationary (in this case differenced), prewhitened (autocorrelation-free) TS.

### Detecting Shift Patterns in the Time Series

In addition we studied the possibility of synchronous climate driven shock signals (structural breaks, shifts, jumps etc) potentially visible in the herring recruitment data as well as in the climate related time series. In contrast to cross-correlations this is rather focussed on the qualitative nature of the climate induced forcing. To investigate this we used the shift detection algorithm according to [37] that looks for synchronous structural breaks in corresponding time series based on a structural break model. This structural break model is essentially a regression model of y_t_ (response variable, for instance NAO, AMO, etc.) over t (time), extended by two shift variables combining a pulse (P) intervention at *t_0_* with a step (S) intervention at *t_0_*+1, thus allowing for both an instantaneous “overshoot” as well as a lagged adjustment in the intervention period:

 (5)with according to

 (6) according to

 (7)α_1_, α_2_, β_0_, and β_1_ are regression parameters.

Meanwhile the shift detection algorithm has been successfully applied in different studies [7], [38], [39] and can be summarized as follows: while iteratively moving a potential shift point t_0_ over the TS (by incrementing t_0_ by 1 year each step), using a specifically defined structural break model, per each iteration relevant decision criteria described below are recorded. These results are displayed in a compound diagrammatic illustration that is termed as a “shiftogram” [37]. A shiftogram consists of a set of elementary diagrams (plots) that summarize graphically the results of all relevant decision criteria (quality-of-fit criteria, marginal *p* values) each of which are synchronized over the same time scale. As the shiftogram simultaneously displays all data and outcomes resulting from iteratively searching for potential shocks in the TS, it facilitates interpretation of the results of the iterative screening process for the detection of shifts in the TS. Hence, a shiftogram consists of the following 10 component graphic panels:

- **shiftogram panel 1:** Plot of the TS (time-series),
- **shiftogram panel 2:** Quality-of-fit plot using the corrected Akaikes information criterion (AICC),
- **shiftogram panel 3:** Plot of the empirical first order autocorrelation coefficient of the model residuals (given the particular structural break specification),
- **shiftogram panel 4:** p value of the first order autocorrelation coefficient from shiftogram panel 4 (t test),
- **shiftogram panel 5:** p value of the statistical test of joint significance of all parameters related to the particular structural break specification (F Test),
- **shiftogram panel 6:** Power plot to indicate the risk of false no-warning; the larger the power, the lower the risk of false no-warning (power = 1–β),
- **shiftogram panel 7:** p value of the statistical test of the pure impulse (F test),
- **shiftogram panel 8:** p value of the statistical test of a break in slope (F test),
- **shiftogram panel 9:** p value of the statistical test of identical levels before and after the shock (ANOVA, F test),
- **shiftogram panel 10:** p value of the statistical test of the variances before and after the shock (Levene-s test on homoscedasticity).

To detect the shift, panels 2, 5 and 6 aid in localizing the position of the change in the time series temporally. All other panels may be consulted in helping to characterize the type of the shift and which of the TS features have been changed.

## Results

### Results from Cross-correlation Analysis

Climate can potentially affect herring recruitment at multiple life stages while a combination of direct and indirect effects may trigger recruitment strength. To test for indirect effects mediated through SSB, we examined the cross correlation factors (CCF) between SSB versus all-year AMO, winter NAO as well as BSI with lags of up to seven years (≈25% of the total time span) to seek potential climatic effects using stationary data (2nd order differenced). To test for direct effects the CCF analysis was repeated between log-transformed *R* and each of the three climate variables in a pair wise fashion.

Given different autocorrelation structures, prewhitening was handled slightly differently for the three climate variables. In the case of the full winter NAO TS (1821–2010), 2^nd^, 3^rd^, 4^th^, and 6^th^ order autoregressive components were fitted to both the 2^nd^ order differenced winter NAO and the *R* and SSB data series, respectively. For the winter BSI TS (1970–2010), 1^st^ and 4^th^ order autoregressive components were fitted to both the differenced winter BSI and the *R* (SSB). For the full all-year AMO TS (1856–2010), 1^st^ and 2^nd^ order autoregressive components were fitted to both the differenced AMO and the *R* (SSB); data series; no moving-average components were significant in either case. Bartlett confidence intervals were used to set confidence limits [29].

Taking the differences clearly leads to mean and variance stationary data, as a comparison of the three left panels of Figure 2 (original data) with its three right ones (differenced data) shows: all statistical tests (slope tests, Levene-s tests) reveal that none of the differenced TS show either a significant trend (*p*<0.05) or heteroscedasticity (*p*>0.1).

**Figure 5:**
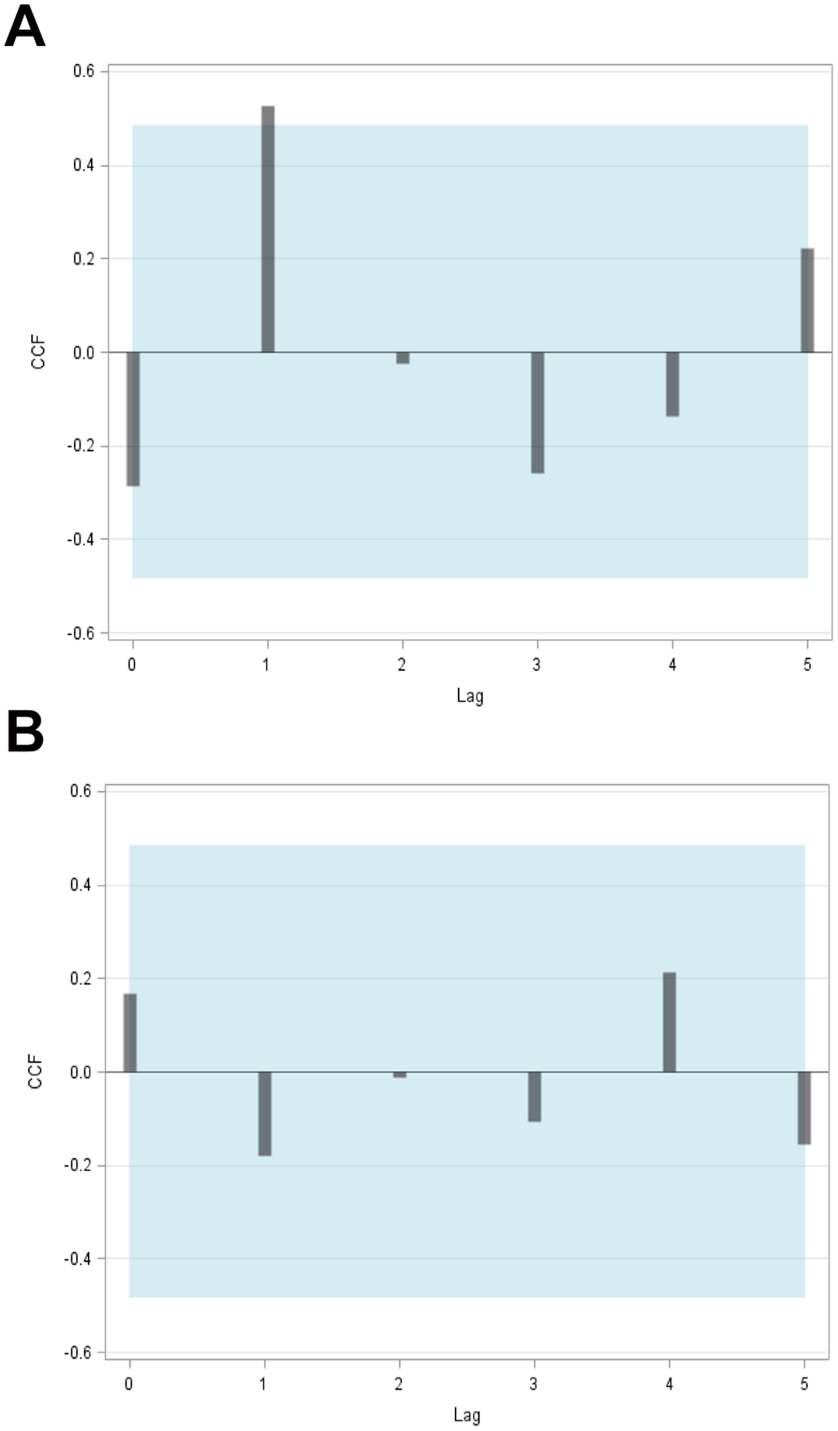
Diagram showing the cross-correlations of (A) detrended and prewhitened log_e_(*R*) plotted against winter BSI as a predetermined variable (with 95% confidence bands shown as the blue shaded area), and (B) detrended and prewhitened log_e_(SSB) against winter BSI. The only significant spike exceeding the 95% confidence bands (blue areas) occurs in the case of log_e_(*R*) in panel (a) at lag 1, indicating a delayed climate effect on herring recruitment.

While the results indicate a strong positive winter BSI signal at lag 1 year when cross-correlated with *R* (Figure 5a), no significant effect can be found on SSB (Figure 5b). The positive winter BSI signal at lag 1 year remains stable when the BSI is aggregated not only over the winter months but the full 1^st^ half of the year (January–June); it however disappears when BSI is aggregated over the full 2^nd^ half of the year (July–December). This feature holds even if the significance level is halved according to splitting the year into two halves to take into account the Bonferroni problem. On the other hand, when disaggregating the winter BSI simply by using monthly indices indicates the strongest signal on *R* to be in February the year before.

In contrast to this winter NAO as well as all-year AMO do not exhibit any significant lags neither on *R* nor on SSB. Hence, the results indicate no statistically significant winter BSI effect on the parent stock why the inclusion of SSB as a structural constraint into the model is statistically uncritical (redundancy, multi-collinearity, variance inflation). Accordingly, climate effects potentially mediated by SSB and log_e_(SSB), respectively, can be ignored.

### Results from Fitting an Extended Cushing-type Stock–recruitment Model

**Figure 6:**
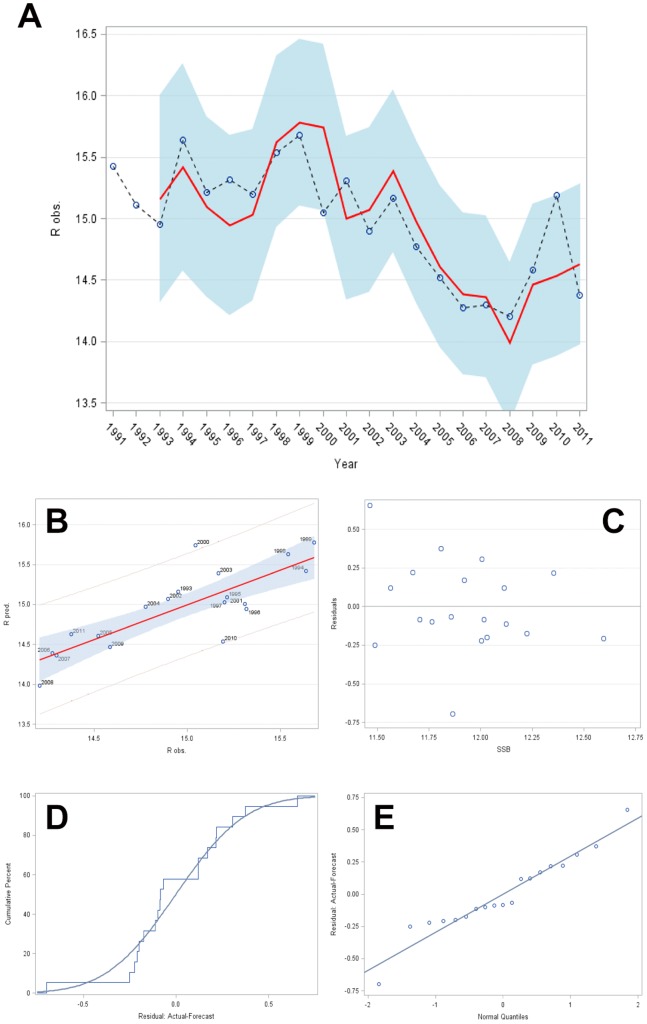
IMAX model panel plot with *R_obs._, R_pred._* and *SSB* on a log-scale. (A) Plot of observed and fitted log_e_(*R*) values of the IMAX recruitment model over time (years) with light blue prediction band (*α* = 0.05). (B) Plot of observed against predicted log_e_(*R*) values indicating good correspondence (*r* = 0.82, *p*<0.0001) with ∼67% of the recruitment variation explained by winter BSI and parental effects. The inner solid line represents the linear correspondence line, the two outer solid lines the 95% confidence interval related to the correspondence line, and the grey area the conic 95% forecast bands related also to the correspondence line. (C) IMAX model residuals plotted against log_e_(SSB) with non-systematic concentrations around the zero line. (D) Explorative distribution function (EDF) of the IMAX model residuals (stepped curve) fit by the cumulative distribution function (CDF) of the normal distribution (continuous line). (E) Q–Q plot of the IMAX model residuals assuming normality.

Given our findings from cross-correlation and the underlying 1^st^ order integrated recruitment and climate processes, we finally identified the following structure of the Cushing-type stock–recruitment model as a predictive generalized transfer function being extended by winter BSI (lagged by one year) (but see also Figure 6):

 (8)where the term (1–*B*) indicates that 1^st^ order differences have been taken for all variables, *µ* is the mean term (corresponding to log_e_(α) in our original model), *γ*_i_ (*B*) the *i*th numerator polynomial of the transfer function for winter BSI and *δ* is the 2^nd^ numerator of the transfer function for SSB. The noise term is given by , with *ε_t_* being the independent random error, and (1–*B^d^*) = (*y_t_–y_t–d_*) a differentiation parameter of order *d* = 0, 1, 2, … years. In short form, the model may be written as IMAX (BSI*_t_*_–1_, log_e_(SSB*_t_*_–1_); *q* = 2, *d = *1). The estimated coefficients are *μ* = 0.09563, *γ*_0_ = 0.66767, *γ*_1_ = −0.67281, *γ*_2_ = −0.31405, *δ = *1.01087, and *φ*_1_ = −0.81471.

The good fit of the IMAX model represented by equation (6) is demonstrated by a high correlation between predicted and observed values of *R* (Figure 6b) (*r*_performance_ = 0.82, *p*<<0.05, *n*_observations_ = 21, *n*_residuals = _19_,_ *k = *2) with AICC = -47.7122 and SBC = -46.2898 representing the lowest values; besides the data point in 2010 no value exceeded the 95% forecast interval (Figure 6a). The strong correspondence is equivalent to explaining about 67% of the recruitment variance by parental effects (SSB) combined with climate (BSI) based on the IMAX model. With the inclusion of winter BSI and by explicitly considering an autocorrelation of 2^nd^ order (q = 2) in the log recruitment residuals, the residuals became normal (Figure 6d,e; *W* ^2^ = 0.0744, *p_W_* ^2^>>0.10; *A* ^2^ = 0.4510, *p_A_* ^2^>>0.10) and uncorrelated (cross checks and autocorrelation checks of the residuals revealed no significant test statistics, with all *p*-values>>0.1), indicating that no other exogenous systematic process is still inherent and as such detectable. Moreover, the model residuals were also homoscedastic (Figure 6c).

In contrast the simple Cushing-type recruitment model (Figure 3) of equation (1) does not appear to be significant (F = 1.23, p>>0.05); this is confirmed by a rather low performance of this model (*r*_performance_ = 0.25, *p*>>0.05, *n*_observations_ = 21, *n*_residuals = _19_,_ *k = *2) with values of AICC = −30.4699 and SBC = −29.0475 exceeding the corresponding values of the IMAX model (*R* on the log scale). The poor correspondence is equivalent to about only 6% of the recruitment variance being explained by parental effects (SSB) based on the Cushing-type recruitment model.

In summary, extending the conventional Cushing-type recruitment model and turning it into a rational transfer function, among others by making use of equations (2), (3) and (6), by pre-whitening and cross-correlating the input and output data series, by including a moving average (MA) component with q = 2, and by shifting back the predicted winter BSI by 1 year, let increase the level of explanation by more than 10 times, i.e. by 61% points (from 6% to 67%).

### Results from Studying Shift Patterns in the BSI and Herring Recruitment Time Series

Figure 6 illustrates the two corresponding shiftograms of (a) the winter BSI running from 1970 to 2010 and (b) the WBSS recruitment time series running from 1992 to 2011 where the black broken vertical lines indicate years of potential structural breaks (shifts) and the three shiftogram panels 2, 5, and 6 (from top) indicated by encircling rectangles (red broken lines) the three major shift detection criteria (AICC, p-joint, power panel). From the (a) shiftogram it is obvious that a rather strong shift occurred around 1989 in the BSI time series. This shift corresponds well with observations of other authors [38], [39], [7]. However, given the different length and starting dates of the two corresponding time series it is obvious that during the overlapping time span of years 1992 to 2010 two rather weak shifts only occurred in the WBSS recruitment time series (being centred around years 1994 and 1999), but without synchronous shock signals in the BSI time series. Thus in summary during the overlapping time period the shiftogram analysis did not reveal any corresponding shock signals in both time series so that the influence can be concluded not being driven by qualitative shocks from climate forcing (such as jumps or other types of shifts in the winter BSI time series that may have led to entire regime shifts through global forcing).

## Discussion

**Figure 7:**
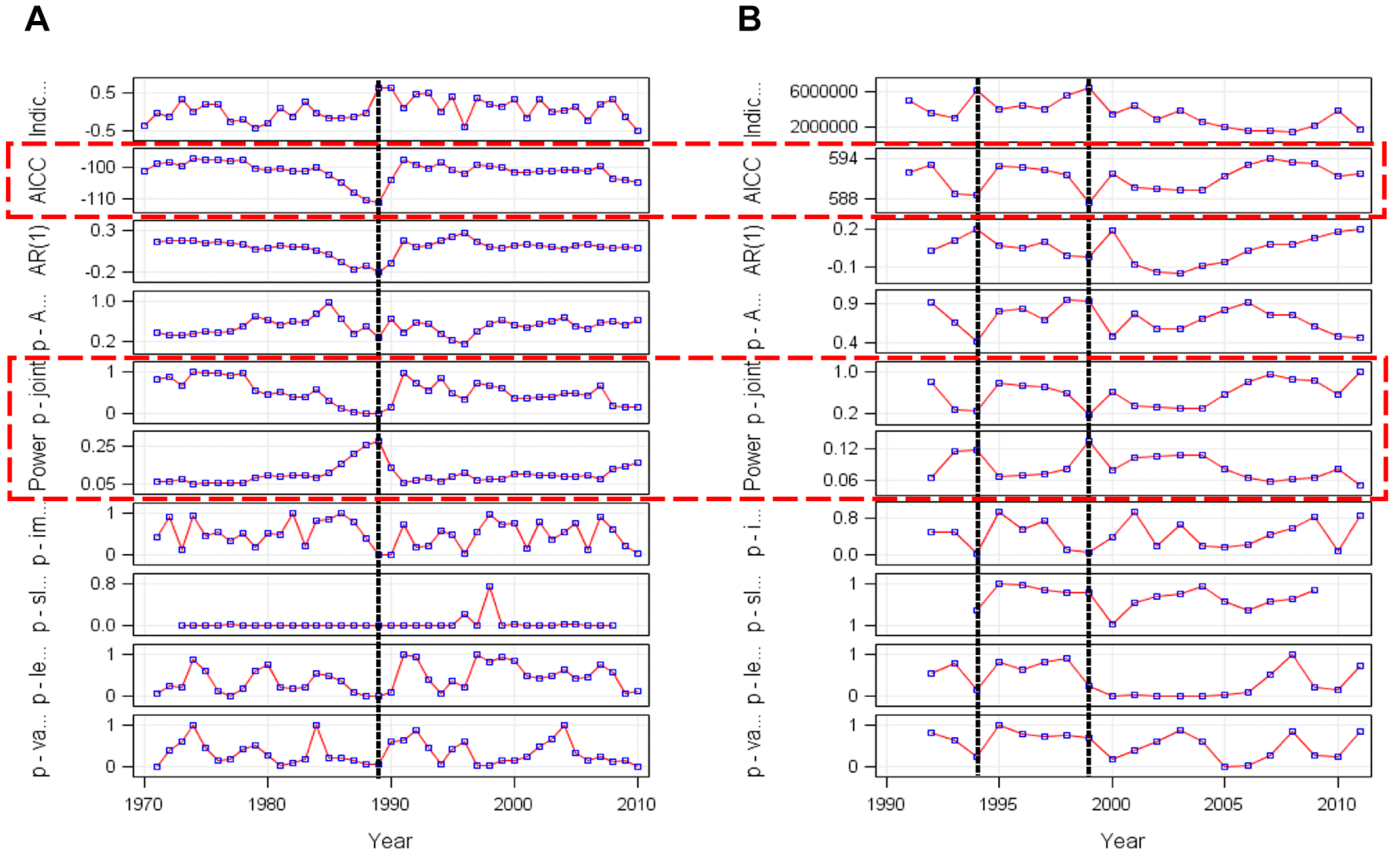
Shiftograms of (A) the BSI and (B) the WBSS recruitment time series. The broken vertical lines indicate years of potential structural breaks (shifts), the open rectangles with broken lines encircle the three major shift detection criteria (AICC, p-joint, power panel).

To detect potential climatic effects we studied the effects of winter NAO (December–March) and winter BSI on WBSS herring recruitment. However, in case of the AMO we used the annual average as in contrast to the monthly NAO indices the monthly AMO cycles do not differ between months [26]. Cross-correlation results suggest that environmental conditions related to the winter BSI but not to winter NAO or all-year AMO are largely responsible for the variability in *R*. This strong winter BSI signal which appears with a response delay of 1 year on *R* persists even when aggregating the BSI index over the 1^st^ half of the year or dissolving the winter effect into monthly effects where the major effect seems to occur in February. The 1 year delay however reflects that the BSI influences WBSS herring recruitment indirectly and at earlier life stages. It is most likely that early life stages in the Greifswalder Bodden retention area are BSI affected as these are usually strongly influenced by abiotic factors. However, a mediating SSB effect can clearly be excluded as the cross-correlation results of BSI with spawning stock biomass does not indicate a BSI influence on recruitment variability via SSB. Moreover, using the new shiftogram method a strong shock-like BSI effect as being visible end of the 1980s (Figure 7a) could not be detected during the overlapping time span thereafter (Figure 7a,b), neither in the BSI nor in the WBSS recruitment time series: The two rather weak shifts appearing in the WBSS recruitment time series do not correspond to the BSI series and thus will clearly have a different reasoning. However, as the relationship between BSI and WBSS recruitment is rather strong on a metric scale future climate shocks may prospectively affect WBSS recruitment also rather strongly. Because the cross-correlation analysis detects those relations with temporal delay, future effects might not immediately become obvious.

A superior effect of climate induced SST has been related to the residuals from a Ricker stock–recruitment curve for Atlantic cod (*Gadus morhua*, L.) [40]. Similar analyses have been conducted using the North Atlantic Oscillation (NAO) as the environmental covariate [41], [42], [43], [44], demonstrating that inclusion of NAO significantly increased explanatory power of stock recruitment models for Northeast Atlantic cod.

This is in line with Post [45] who discussed delayed processes with regard to climate, with specific focus on the relationship between NAO and other regional environmental variables and marine populations in the North Atlantic, concluding that “lagged population responses to large-scale climatic variability may arise when the proximal abiotic factor influencing the population dynamics is itself correlated with regional atmospheric processes at some time in the past”. Lehmann *et al.* [15] investigated that the large-scale atmospheric conditions over the North Atlantic are correlated with regional atmospheric conditions over the western Baltic, but e.g. the NAO-index only accounts for 25% of the variance of the sea level pressure anomaly over the western Baltic (BSI). In contrast to the NAO-index (ca. 3000 km) the BSI represents a much smaller meridional atmospheric air pressure gradient (600 km), i.e. the BSI includes the gradients of synoptic-scale air pressure gradients which are not represented by remote large-scale atmospheric forcing patterns. This was already observed by Osborn *et al.* [46], who found correlation coefficients between 0.3 and 0.6 for the Baltic Sea area. The local atmospheric conditions have a strong influence on many environmental processes in the Baltic Sea. A significant correlation of inter-annual changes in the reproduction volume of eastern Baltic cod and SST with changes in the BSI during winter months has been demonstrated by a studies performed by Hinrichsen *et al.* [16], [17], while BSI-values obtained during spring and summer have shown a strong impact on eastern Baltic cod larval and juvenile distributions [47].

In general, metabolism and physiology of boreal fish species are very sensitive against changes of temperature regimes [48]. Recruitment models with explicit consideration of environmental factors have been widely applied to various fish stocks to examine the possible influence of temperature on recruitment e.g. [49], [41], [25], [26], [50]. The survival of early herring life stages in particular has been demonstrated to be especially vulnerable to shifts of oceanographic temperature regimes [51]. For Pacific herring (*C. pallasii*, Valenciennes 1847) interannual variability of recruitment was shown to be strongly correlated with climatic indices affecting coastal upwelling in the proximity of Northeast Pacific estuaries [52]. This indicates that regional climate indices indeed explain the variability of the temperature regime as a baseline for sensitive ecological cascades relying on a suite of interlinked mechanisms. As postulated by Hjort [53] and refined by Cushing [54], the survival of larval fish is determined by the critical period when larvae finished the yolk reservoir and start feeding actively. If there is less suitable planktonic prey available at this point of “first feeding” larvae will inevitably starve. Therefore early life stage mortality is widely depending on match-mismatch events of appearance of larvae and seasonal plankton blooms [54], [55]. The timing of both components underlies interannual shifts that might well be explained by regional climatic variability. Induced by climatic forcing a regime shift was documented for the Baltic Sea concerning important changes in the prey composition of e.g. herring [56]. The relation among climate indices and the trophic regime of the outer Baltic Sea is therefore rather a tested hypothesis than an assumption. Investigating drivers of recruitment variability in various Baltic Sea herring stocks, Cardinale et al. [57] indicated BSI to be a strong predictor of WBSS herring recruitment strength. Since the base line for the authors’ explanatory approach was a spawning period from January to March a direct linkage of winter BSI temperature regimes on spawning process and early herring ontogeny seems plausible. However, the peak spawning period of the Rügen herring stock component is located in late March to mid-May [58] and therefore a direct temperature effect of winter BSI on spawning and egg development is rather unlikely. A direct effect of winter BSI on larval metabolism and -growth can most likely also be rejected due to seasonal decoupling of larval peak abundance and temporal range of winter BSI, even the BSI effect persists over the 1^st^ half of the year as our study showed; but is also indicated that the major signal occurs in February and hence one month earlier than the start of the spawning activities in this region. However, indirect cascading effects of winter climate variability on egg survival and available prey fields for early larval stages according to match-mismatch effective in coastal inshore systems and transitional waters of the Baltic Sea might represent likely mechanisms involved in climate induced recruitment variability of WBSS herring. However, as the BSI effect is only small for the 2^nd^ half of the year it is not very likely that recruitment variations are associated with wind-induced advective transport mechanisms of late larval and juveniles stages towards their nursery grounds. Our findings on effects of climate forcing on WBSS herring correspond well with similar results on other clupeid species in different ecosystems regarding fish recruitment to be predominately driven by physical processes [59], [60], [61].

Unlike other important commercial fish species of the Baltic Sea such as cod and sprat (*Sprattus sprattus*, L.), herring larvae hatch from demersal eggs attached to benthic substrates [62]. Therefore they are not dispersed by current regimes potentially transporting eggs to more favourable habitats if conditions in the spawning grounds become unsuitable. Additionally herring eggs are not capable of vertical adjustment to certain stratified water layers by mass specific buoyancies [63], [64]. Winter BSI however will determine climate condition in Baltic Sea transitional waters during the early herring spawning period and thus affect the immediate physico-chemical environment on the particular spawning beds eventually affecting hatching success. Since early spawning cohorts occur in March, their embryonic development is impacted by climate conditions and thus may be affected by winter BSI. However, very often in practice egg or early larval-abundances are used as indices or proxies for the abundance of the parental stock e.g. [7], [65] while large-larvae abundances are used to approximate recruitment e.g. [7]. The reason is that the relationship between SSB and abundance of eggs as well of small larvae in the water appears to be approximately linear. Therefore we can exclude a direct BSI effect on egg development or egg survival of WBSS herring as no significant linear cross-correlation signal of BSI on SSB was detected.

As demonstrated for Baltic sprat, simple correlation analysis is suitable for detecting long-term trends and a general relationship between physical variables and recruitment [66]. However, the power of simple correlation analysis is limited to symmetric and temporally corresponding processes. Therefore it may fail to detect delayed responses and causalities. For instance, a global scale effect such as climate forcing may need some time before penetrating through an entire causal chain because of cascading or accumulating effects with intermediate processes being involved [26], [37].

On a global scale climatic drivers can be diverse and slight changes may lead to entire regime shifts since multiple ecosystem components are affected synchronously. These effects may be quantitative (expressed by correlations on a metric scale) or qualitative (non-metric scale; corresponding shock signals, regime shifts). Climate forcing on marine resources is evident in long-term records of fishery and fish abundance and also in palaeo-ecological and related observations [67], [68]. Changes of distribution and marine fish productivity under changing environmental conditions have now widely been documented [69], [70]. Hence, the need to account for shifting climatic conditions by adjusting fishery management reference values is increasingly being acknowledged [71], [50], [72]. Synergistic effects of climate change and harvesting can alter the resilience of exploited species and influence long-term sustainability [73]. Integrating these findings into stock assessment models – along with exploitation patterns – can be expected to result in a significantly improved WBSS management.
